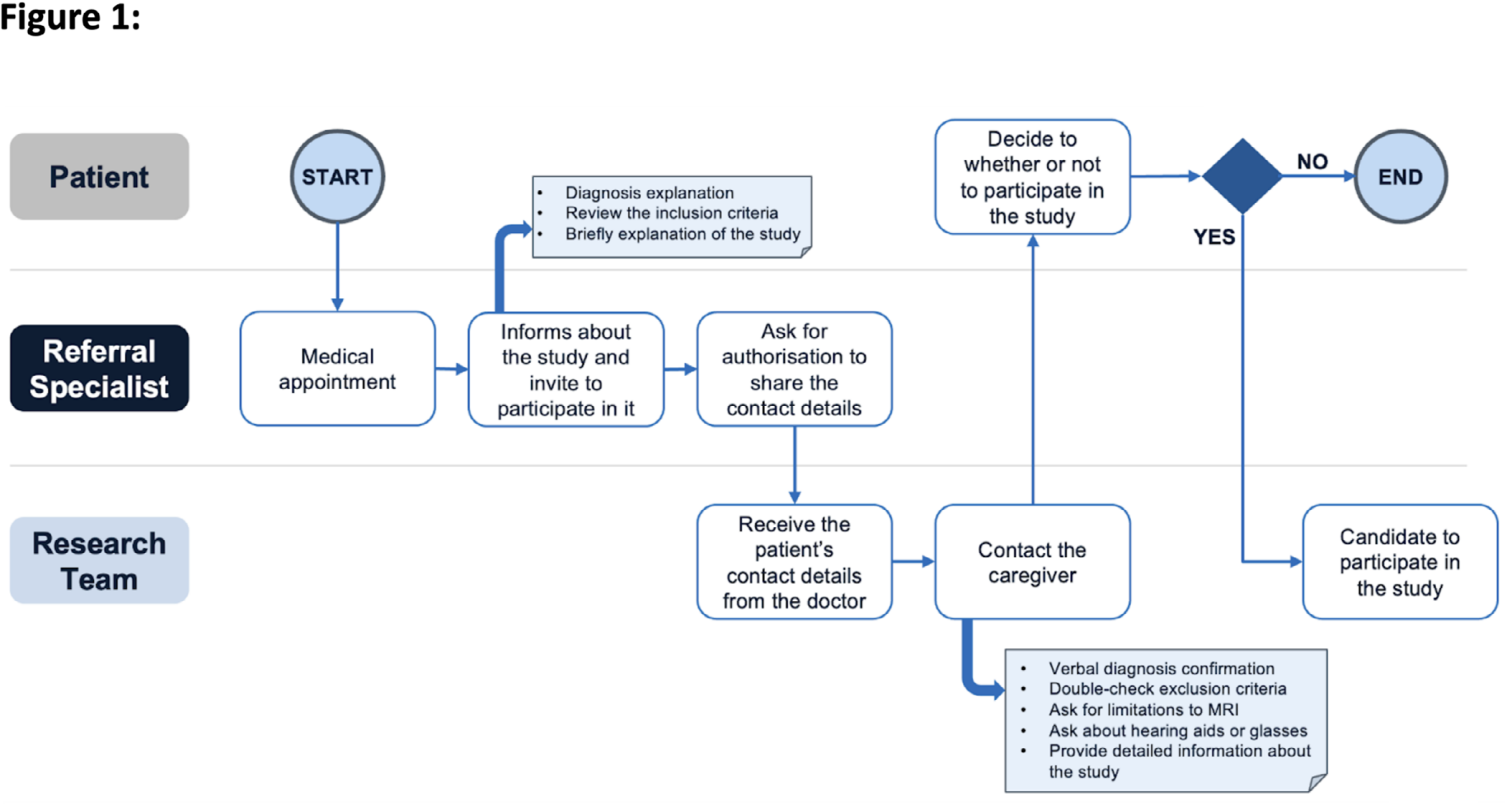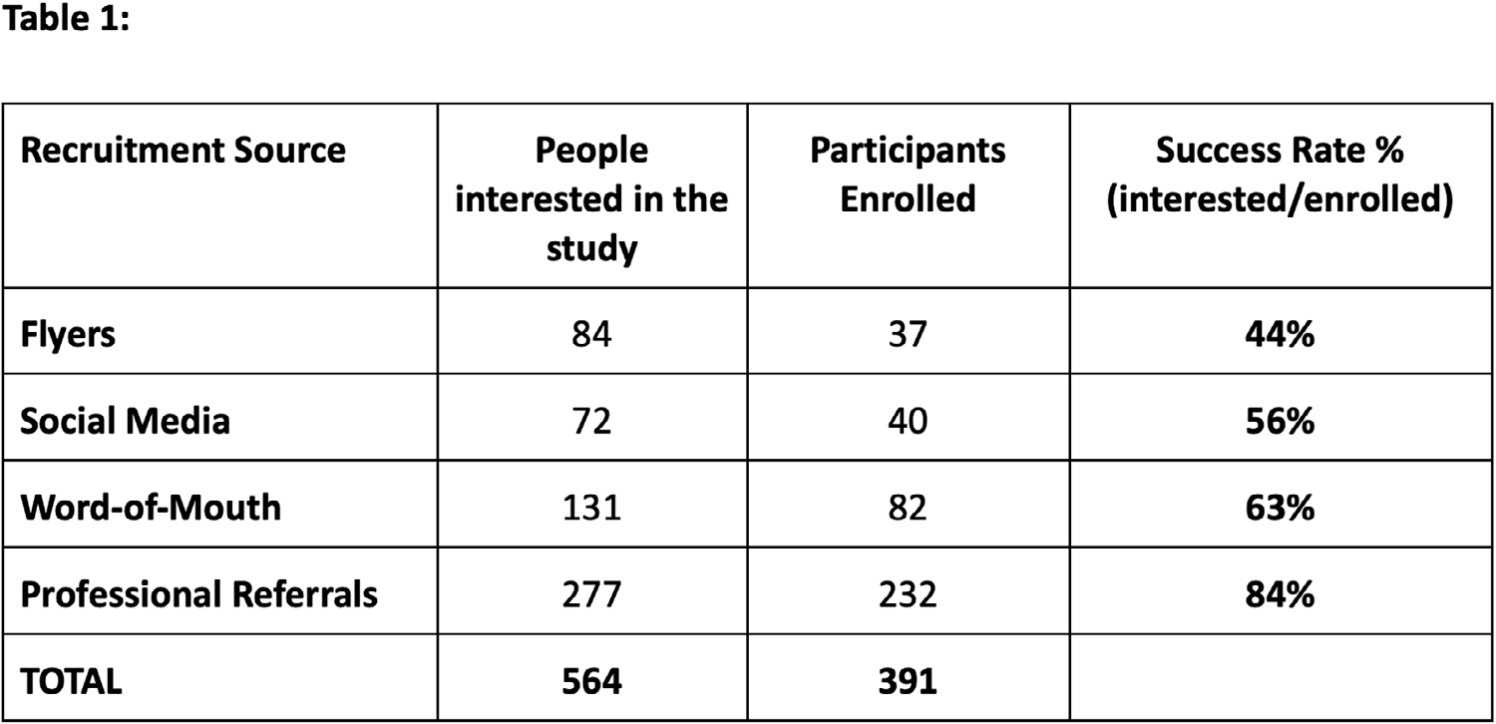# Effective recruitment strategies for engaging the Peruvian population in the ReD‐Lat study

**DOI:** 10.1002/alz70860_104080

**Published:** 2025-12-23

**Authors:** Belen Custodio, Rosa Montesinos, Maria Eugenia Godoy, José Carlos Huilca, Diego Bustamante‐Paytan, Diego Chambergo‐Michilot, Katherine Agüero‐Flores, Graciet Verastegui, Marco Malaga, Karol Melissa Lipa‐Pari, Nilton Custodio

**Affiliations:** ^1^ Unidad de Investigación de Deterioro Cognitivo y Prevención de Demencia, Instituto Peruano de Neurociencias, Lima, Lima, Peru; ^2^ Unidad de Investigación y Docencia, Equilibria, Lima, Peru., Lima, Lima, Peru; ^3^ Latin American Brain Health Institute (BrainLat), Universidad Adolfo Ibáñez, Santiago, Región Metropolitana de Santiago, Chile; ^4^ Cognitive Neuroscience Center (CNC), Universidad de San Andrés, Buenos Aires, Buenos Aires, Argentina; ^5^ Unidad de Investigación de Deterioro Cognitivo y Prevención de Demencia, Instituto Peruano de Neurociencias, Lima, Peru, Lima, Lima, Peru; ^6^ Universidad Científica del Sur, Lima, Perú, Lima, Lima, Peru; ^7^ Hospital Nacional Cayetano Heredia, Lima, Lima, Peru

## Abstract

**Background:**

Recruitment for dementia research remains a persistent challenge, particularly when trying to reach underrepresented groups such as Latin Americans with traditional enrolment strategies. This abstract describes the experience of the Research Department at the Instituto Peruano de Neurociencias in employing culturally tailored strategies to enhance participant recruitment in Lima, Peru. Additionally, it quantifies recruitment sources as part of the ReD‐Lat study.

**Method:**

This retrospective study outlines the strategies used to recruit participants for the ReD‐Lat study between 2023 and 2024. ReD‐Lat is a multi‐partner consortium aimed to expand dementia research in Latin America.

Participants were recruited from the following sources: (1) flyers, (2) social media, (3) word‐of‐mouth, and (4) professional referrals. The flyers included study details and contact information and were distributed in churches and healthcare centers. Recruitment materials were also disseminated through paid Facebook advertisements. All flyers and social media posts were written in Spanish. Word‐of‐mouth recruitment was defined as referrals from individuals who had already participated in the study and shared their experiences with others outside the study. Professional referrals involved presenting the project to physicians, who then referred patients to the research department. This referral process is detailed in Figure 1. Screening began when potential participants either contacted the research team directly or were reached out to by team members. During the first contact, participants were asked how they learned about the study. Their responses were recorded in an anonymous database and subsequently categorized into one of the predefined recruitment sources mentioned above.

**Result:**

From January 2023 to December 2024, a total of 564 participants were interested in the ReD‐Lat study and contacted the Research Department, from which 69% (391) were enrolled. Success rates for each recruitment source are shown in Table 1.

**Conclusion:**

Tailored recruitment strategies, particularly professional referrals and word‐of‐mouth, demonstrated high success rates in enrolling participants for the ReD‐Lat study in Lima, Peru. These findings highlight the importance of leveraging community trust and professional networks to improve recruitment outcomes for dementia research in underrepresented populations.